# A 12-week lifestyle intervention: effects on fatigue, fear, and nutritional status in children with a Fontan circulation

**DOI:** 10.3389/fped.2023.1154015

**Published:** 2023-11-06

**Authors:** L. E. Scheffers, W. A. Helbing, T. Pereira, S. Walet, E. M. W. J. Utens, K. Dulfer, L. E. van den Berg

**Affiliations:** ^1^ Department of Paediatrics, Division of Paediatric Cardiology, Erasmus MC Sophia Children’s Hospital, Rotterdam, Netherlands; ^2^ Department of Paediatrics, Division of Gastroenterology, Erasmus MC Sophia Children’s Hospital, Rotterdam, Netherlands; ^3^ Respiratory Medicine and Allergology, Department of Paediatrics, University Medical Center, Erasmus MC Sophia Children’s Hospital, Rotterdam, Netherlands; ^4^ Department of Paediatrics, Center for Lysosomal and Metabolic Diseases, Erasmus MC, Rotterdam, Netherlands; ^5^ Division of Dietetics, Department of Internal Medicine, Erasmus MC, Rotterdam, Netherlands; ^6^ Department of Child and Adolescent Psychiatry/Psychology, Erasmus MC Sophia Children’s Hospital, Rotterdam, Netherlands; ^7^ Research Institute of Child Development and Education, University of Amsterdam, Amsterdam, Netherlands; ^8^ Department of Child and Adolescent Psychiatry, Amsterdam University Medical Center/Levvel, Amsterdam, Netherlands; ^9^ Intensive Care Unit, Department of Paediatrics and Paediatric Surgery, Erasmus Medical Centre Sophia Children’s Hospital, Rotterdam, Netherlands; ^10^ Department of Orthopedics and Sports Medicine, Erasmus MC, Rotterdam, Netherlands

**Keywords:** Fontan circulation, lifestyle intervention, fatigue, fears, body composition, rest energy expenditure

## Abstract

**Introduction:**

Children and adolescents with a Fontan circulation are less physically active compared to healthy peers. In the current study, effects of a 12-week lifestyle intervention on fatigue, fears regarding exercise, caloric intake, rest energy expenditure (REE), and body composition were measured in children with a Fontan circulation.

**Methods:**

This study was a semi-cross-over randomized controlled trial. The lifestyle intervention consisted of a 12-week high-weight resistance training (three supervised training sessions a week) supported by high-protein diet (>2 g/kg) and tailored recommended caloric intake. Fatigue (measured by the validated PedsQol Multidimensional Fatigue Scale), fears regarding exercise (measured on a fear thermometer), REE (measured using indirect calorimetry), caloric intake and body composition using air displacement plethysmography, and four-skinfold method were measured before and after the intervention and control period.

**Results:**

Twenty-seven pediatric Fontan patients, median age 12.9 years (IQR: 10.5–16.2), of the included 28 patients successfully completed the program. Before training, both child- and parent-reported levels of fatigue were significantly worse on all domains (general, sleep/rest, and cognitive fatigue) compared to healthy peers. After training, parent-reported fatigue significantly improved on the general and cognitive fatigue domains [effect size +16 points (7–25), *p* < 0.001, and +10 points (2–17), *p* = 0.015, compared to the control period]. Before training, fear regarding exercise scored on the fear thermometer was low for both children and parents (median score 1 and 2, respectively, on a scale of 0–8). After training, child-reported fear decreased further compared to the control period [effect size −1.4 points (−2.3 to −0.6), *p* = 0.001]. At baseline, children had increased REE +12% compared to reference values, which did not change after exercise. Children ate an average of 637 calories below recommended intake based on REE, caloric deficit became smaller after the intervention, and protein intake increased compared to the control period [−388 calories (−674 to −102), *p* = 0.008, and +15 g (0.4–30), *p* = 0.044]. Body fat percentage did not change significantly.

**Conclusion:**

A 12-week lifestyle intervention improved parent-reported fatigue symptoms in the children, further decreased child-reported fears, and increased caloric and protein intake.

## Introduction

Treatment of patients born with a single functional ventricle consists of a series of operations, leading to a Fontan circulation (1). Due to improvements in pre-, peri-, and post-operative care, this population is growing, and an increasing number of patients reach adulthood. In adulthood, increased risk of obesity and acquired cardiovascular disease in this population becomes more important (1–4). Regular physical activity is one of the most important contributors to prevent aforementioned morbidities, and also has numerous other health benefits, including higher energy levels and less fatigue (5). Several studies investigating physical activity levels in children with a Fontan circulation showed decreased physical activity levels and sports participation compared to healthy peers (6, 7). Multiple reasons for lower sports participation in children with congenital heart diseases (CHDs) have been identified, including fatigue, parental (and sport instructors) overprotection, fear regarding exercise, and (unnecessary) restricting physician recommendations (8–10). The study by Moola et al. found that fatigue, fear, and exclusion (not being able to perform on the same levels as healthy peers) further decreased the value children with a congenital heart disease ascribed to sport and physical activity (10). A systematic review published by our group showed that physical training in Fontan patients may lead to improvements in physical health and quality of life, with none of the included studies reporting negative outcomes related to the exercise programs (11). Interestingly, only one study investigated training effects on fears and none looked at exercise effects on fatigue, an important limiting factor in chronically ill children (12–14). We, therefore, investigated the effects of a lifestyle intervention on fatigue, fears regarding exercise, rest energy expenditure (REE), nutritional intake, and body composition in children with a Fontan circulation.

## Methods

This was a semi-cross-over randomized controlled trial investigating effects of a 12-week lifestyle intervention on children with a Fontan circulation. This trial was conducted between December 2020 and July 2022 at the Department of Pediatric Cardiology, Erasmus MC Sophia Children’s Hospital, Netherlands. The study was performed in accordance with the Declaration of Helsinki. It was approved by the local Ethics Committee of Erasmus MC (NL.70912.078.19) and registered at the Dutch trial register (https://trialsearch.who.int/Trial2.aspx?TrialID=NL8181) as Trial NL8181. A detailed protocol of this study has been published previously (15).

### Participants

Children with univentricular heart defects, who had a completed series of interventions resulting in the Fontan circulation, aged 6–18 years, were eligible for enrollment. Physical inability to perform a cardiopulmonary exercise test (CPET), participation in other exercise training programs, and contra-indications for exercise (a ventricular obstruction of >60 mmHg and arrhythmias) were exclusion criteria. All patients were recruited at the outpatient clinic of the Erasmus MC Sophia Children's Hospital. Signed informed consent was obtained from all participants and/or parents before enrollment.

### Study design and intervention

**Figure 1:**
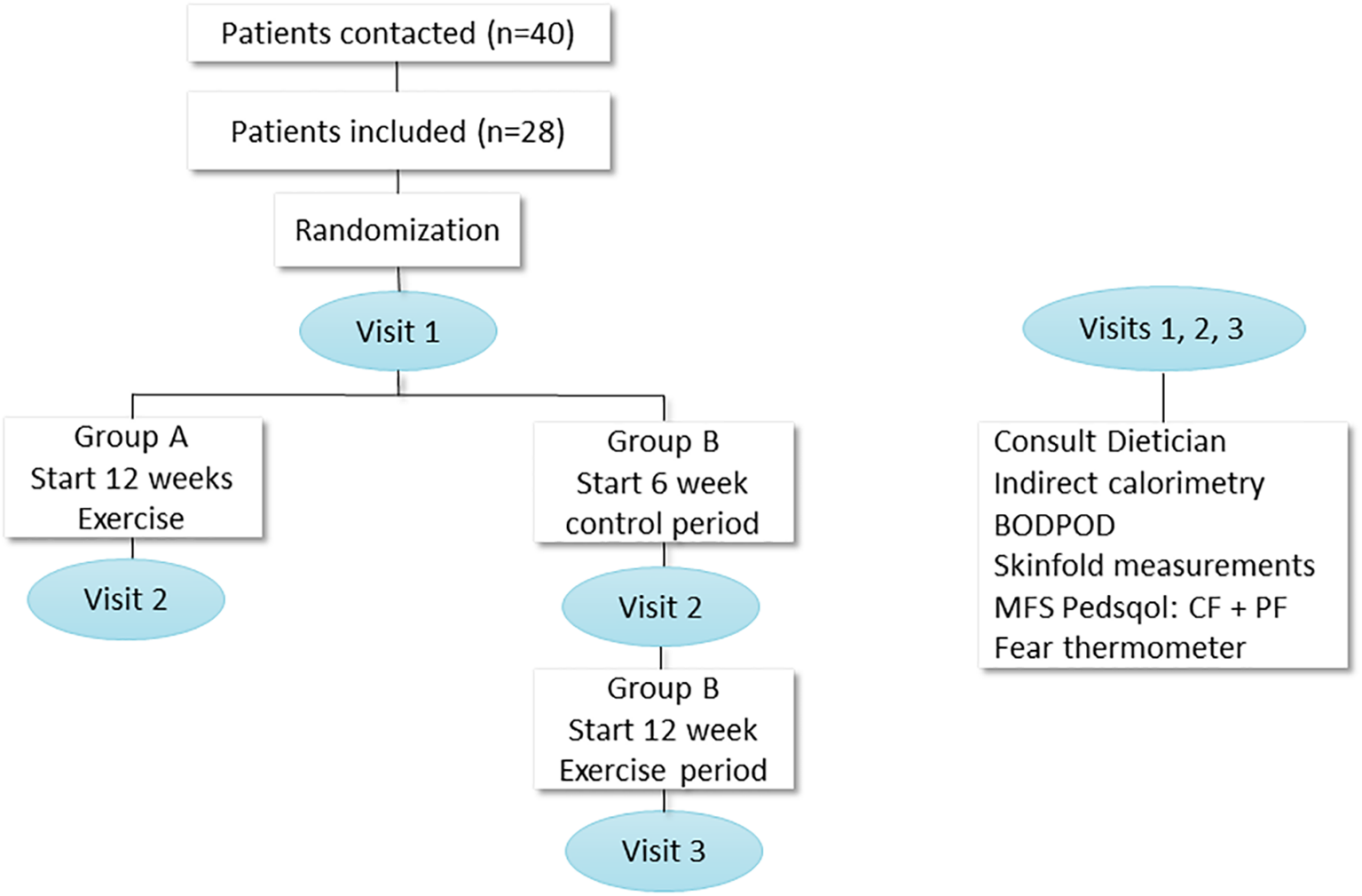
Study design with measurements. N, number; PedsQol, pediatric quality of life; MFS, multifatigue scale; CF, child form, PF, parent form; BODPOD, body composition measurement system.

Figure 1 shows the study design, visits, and measurements. Randomization was performed in blocks of four or six using Castor (Clinical electronic Data Capture) (16). All children were randomized in group A (start exercise) or group B (start control period, duration 6 weeks). Group A started the intervention immediately after the first assessment, and group B started after a period of 6 weeks during which they received “care as usual” (thus this 6 weeks period of group B serves as the control period for all patients with a 2:1 ratio). Each study visit consisted of two assessment days with at least 3 days and maximally 7 days in between during which patients continued normal daily life. The tailored lifestyle intervention was designed as previously described in our trial design paper (15). The tailored individual physical training program lasted 12 weeks and consisted of three supervised training sessions per week (supervision performed by a trained physiotherapist close to participant's home) lasting 45 min each. The standardized training program started with 10 min of walking on a treadmill with maximal incline, rowing, or cross trainer exercise, where after children performed progressive overload resistance training focused on the leg muscles. Each exercise was performed in three sets of six to eight repetitions. Exercises included squats, (single) standing/seated calf raises, hip trusts, step-ups, leg press, leg abduction, and leg adduction. The full training program can be found out in other study (17). In order to support muscle growth, patients received a high-protein diet of 2 g/kg per day and a recommended caloric intake per day, total energy expenditure (TEE) (calculated using the Schofield formula and based on measured REE and corrected for weight) including a brochure regarding healthy diet in children (designed by the “Voedingscentrum,” the Dutch government–supported nutritional center) (18). LES visited all first training sessions and a training (either live or via video connection) of each patient every 2 weeks, to monitor uniform execution of the training program. During the intervention, LES telephoned patients weekly to monitor safety, side effects, and assure compliance for both the training and dietary advice.

### Outcome measurements

The outcomes of the 12-week lifestyle intervention were fatigue, fear regarding exercise, and nutritional status (body composition, REE, and caloric intake).

#### Fatigue

The PedsQL Multidimensional Fatigue Scale (MFS) age-specific child form (CF) and parent form (PF), consisting of 18 items, divided into three domains, were used to assess fatigue in children/adolescents, with a higher score indicating less fatigue symptoms (19). Baseline outcomes were compared to previously published data in healthy Dutch children (*n* = 366) and their parents (*n* = 497) (20).

#### Fear of exercise

Children and parents had individual semi-structured interviews with a psychologist. They were asked to score fear regarding exercise on the fear anxiety thermometer, 0 (no fears at all) up to 8 (high anxiety) (Supplementary Data S1) (14).

#### Nutritional status

At each visit, patients height and weight were measured, and body composition was assessed using a skinfold caliper (four skinfolds method) and air displacement plethysmography (ADP) on whole-body densitometry using the BOD POD (BOD POD body composition system, COSMED, Ltd, Concord, CA, USA). Body fat percentage (%BF) was calculated using the equations published by Brook, Drunin, Rahaman, and Wormsley et al. for the skinfold measurements, and the Lohman equation for the ADP (21–24). Measured body fat percentages using the skinfold method were compared with a large European reference group (25). As no large reference value studies using BOD POD currently exist in children, fat percentage measured by BOD POD were compared to body fat reference curves for children of McCarthy et al. (26). All patients filled in a detailed food diary for three consecutive days, which was checked by the dietician SW and used to calculate caloric intake and protein intake. Children underwent an indirect calorimetry (Quark-RMR, Cosmed, Italy) performed by the dietician. Measured REE was compared to predicted REE using the Schofield equation (27), whereas REE > 1.1 was defined is hypermetabolism and REE < 0.9 was defined as hypometabolism. TEE is calculated by multiplying measured REE with factors such as activity and growth (28).

#### Sample size calculation and statistical analysis

Power calculation for this study was based on the primary study outcome of change in peak VO2 after the physical training program, and 28 patients with a Fontan circulation were included in this study (15). Data were collected in Castor (Clinical electronic Data Capture) (16), and all analyses were performed using IBM SPSS Statistics 25.0 (IBM Corp, Armonk, NY, USA). Patient characteristics were described using descriptive statistics. Baseline characteristics between groups were compared with the Mann–Whitney *U* and *χ*^2^ test for proportions. All data were analyzed as non-parametric due to the small sample size. Difference between baseline fatigue domains and healthy population were analyzed using the Wilcoxon one sample test. Differences over the exercise period and control period were analyzed using the Wilcoxon signed ranks test for proportions. A generalized equation approach model was used to compare change over the control to the exercise period [described as the effect size including 95% confidence interval (CI) and matching *p*-value] accounting for the correlation of the repeated measurements in the control/active group. The working correlation matrix was set as unstructured. The significance level was determined at *p* < 0.05.

## Results

### Patient characteristics

In total, 40 patients were contacted to participate in the study (17). Reasons not to participate were too busy with school obligations (*n* = 6), parents were unable to bring the child to the hospital/physical therapist (*n* = 3), private circumstances (*n* = 1), already playing sports >3 times a week (*n* = 1), and one patient was scheduled for an operation. A total of 28 patients were included in the study, and 27 patients successfully completed the exercise intervention. The median age of the included patients was 12.9 (10.5–16.2) years, and 37% were female. Patient characteristics can be found in Table 1. Baseline echocardiograms showed a good systolic function in 15 and a moderate systolic function in the other patients. Atrioventricular valve insufficiency was mild in most patients (*n* = 13), moderate in four patients, and trivial or not present in the other patients. Three patients had mild aortic valve insufficiency, and other children did not have aortic valve insufficiency. Children showed no signs of arrhythmia at baseline.

**Table 1:** Patient characteristics.

	Study population (*n* = 27)	Group A (*n* = 13)	Group B (*n* = 14)	*p*-values
Female, *n* (%)	10 (37)	4 (31)	6 (43)	0.516
Age, years	12.9 (10.5–15.7)	12.8 (10.5–15.7)	12.9 (10.5–15.6)	0.792
Age at Fontan completion, years	2.8 (2.3–3.8)	2.7 (2.1–3.9)	3.1 (2.6–3.6)	0.413
Body mass index, kg/m^2^	17.8 (16.8–19.2)	17.2 (16.8–18.7)	18.0 (17.3–19.2)	0.881
Diagnosis, *n*				
Double inlet left ventricle	4	3	1	0.651
Pulmonary atresia	6	2	4	
Tricuspid atresia	3	2	1	
Hypoplastic left heart syndrome	8	3	5	
Double outlet right ventricle	6	3	3	
Dominant ventricle, *n*				
Left	10	4	6	0.707
Right	14	6	8	
Other	3	2	1	
Medication during study, *n*				
Vitamin K antagonist	8	3	5	0.822
Platelet inhibitor	19	10	9	
ACE inhibitor	2	2	0	
Beta-blocker	2	1	1	
Diuretic	0	0	0	
Systolic ventricular function (*n*)				
Good	15			
Moderate	10			
Aortic insufficiency (*n*)				
None/trivial	21			
Mild	3			
AV insufficiency (*n*)				
None/trivial	8			
Mild	13			
Moderate	4			

ACE, angiotensin-converting enzyme; AV, atrioventricular (insufficiency).

Data are presented as median (IQR) or number (*n*)/percentage (%). Difference between group A and B was calculated using the Mann–Whitney *U* or *χ*^2^ test for proportions.

### Compliance and drop-outs

Compliance was high, with a median training session attendance of 33 (32–34) out of 36 sessions. In total, 27 patients completed the exercise intervention. One patient dropped out after the first training session, as his parents were unable to bring him to the training sessions.

### Fatigue

Before training, self- and parent-reported levels of fatigue in children/adolescents were significantly worse on all domains (general, sleep/rest, and cognitive fatigue) compared to healthy children (Table 2) (20). After training, parent-reported fatigue significantly improved on the general fatigue and cognitive fatigue domains compared to the control period [+16 points (7–25), *p* < 0.001, and +10 (2–17), *p* = 0.015]. Self-reported fatigue scores did not significantly improve after training.

**Table 2:** Fatigue measured using the multidimensional fatigue scale and fear of exercise measured using the fear thermometer.

	Exercise period (*n* = 25 children, *n* = 48 parents)			Control period (*n* = 14 children, *n* = 27 parents)					
	Before	After	*p*-values	Before	After	*p*-values	Effects size vs. control period (95% CI)	*p*-value difference exercise vs. control period	Domain score compared to healthy population
Child report									
General fatigue	71 (63–88)	79 (63–94)	0.276	75 (63–89)	71 (64–97)	0.319	5 (−5–15)	0.324	**Decreased** *
Sleep/rest fatigue	63 (52–92)	71 (54–88)	0.175	69 (56–88)	71 (57–79)	0.964	3 (−7–13)	0.580	**Decreased** *
Cognitive fatigue	67 (35–81)	58 (44–85)	0.190	63 (35–75)	67 (33–85)	0.422	5 (−4–15)	0.281	**Decreased** *
Parent report									
General fatigue	69 (54–83)	77 (67–92)	**<0**.001*	71 (67–92)	75 (58–88)	0.165	16 (7–25)	**<0**.001*	**Decreased** *
Sleep/rest fatigue	79 (63–88)	77 (59–92)	0.438	79 (63–88)	79 (71–88)	0.390	1 (−6–7)	0.847	**Decreased** *
Cognitive fatigue	71 (54–83)	75 (71–92)	**<0**.001*	75 (42–96)	71 (57–79)	0.507	10 (2–17)	**0**.015*	**Decreased** *
Fear thermometer									
Fear thermometer score children	1 (0–2)	0 (0–2)	0.148	0 (0–1)	1 (1–2)	**0**.034*	−1.4 (−2.3–−0.6)	**0**.001*	**—**
Fear thermometer score parents	2 (0–2)	2 (0–2)	0.905	2.0 (0.5–4.0)	2 (1.0–2.0)	0.162	0.8 (−0.4–2)	0.186	**—**

Data are presented as median (IQR). Differences over the exercise period and control period were analyzed using the Wilcoxon signed ranks test. A generalized equations approach model was used to compare change over the control to the exercise period (described as the effect size including 95% CI and matching *p*-value). Difference between the baseline MFS outcomes and healthy population was measured using the Wilcoxon one sample test.

Bold and * values mean *p* < 0.05.

### Fears regarding exercise

Before training, parents and children obtained low scores on the fear thermometer [1 (0–2) and 2 (0–2), respectively, on a scale from 0 to 8] (Table 2). After training, child-reported fear decreased significantly compared to the control period [effects size −1.4 points decrease (−2.3 to −0.6), *p* = 0.001].

### Nutritional status

Fontan patients showed normal growth compared to healthy children (Table 3). Body fat percentage did not change after the intervention (Table 3). Body fat percentage measured using the skinfold methods was lower compared to fat percentage measured using the BOD POD [9.1% (6.7–12.9) vs. 16.5% (13.3–22.5)]. At baseline, children had increased REE +12% compared to reference values, which did not change significantly after exercise. Based on measured REE, almost all children did consume too little calories, on average 637 below recommended TEE (=82% of recommended intake). After training, the caloric deficit became smaller and protein intake increased compared to the control period [−388 calories deficit (−674 to −102) = 89% of recommended intake, *p* = 0.008, and +15 g (0.4–30), *p* = 0.044, *n* = 19, as 8 children did not complete their food diary after training].

**Table 3:** Body composition, intake, and REE.

	Exercise period (*n* = 27)			Control period (*n* = 14)				
	Before	After	*p*-values	Before	After	*p*-values	Effects size exercise vs. control period (95% CI)	*p*-value exercise vs. control period
Height (cm)	158 (145–170)	160 (144–168)	**<0.001**	154 (143–167)	155 (145–168)	0.109	0.5 (0.2–1.2)	0.161
Weight (kg)	42 (34–54)	46 (35–56)	**<0**.**001**	41.3 (32–56)	42 (33–53)	0.109	2.5 (−0.4–2.8)	0.096
Height/age (SDS)	−0.4 (−1.3–0.2)	−0.4 (−1.1–0.3)	0.447	−0.5 (−1.5–0.3)	−0.4 (−1.3–0.2)	0.624	0 (−0.2–0.3)	0.664
Weight/length (SDS)	−0.4 (−0.6–0.4)	−0.1 (−0.7–0.4)	0.809	0 (−0.8–0.6)	−0.4 (−0.8–0.2)	0.055	0.2 (−0.1–0.5)	0.209
BMI (kg/m^2^)	17.6 (16.4–19)	17.8 (16.6–19.4)	0.069	17.7 (15.9–18.8)	17.6 (15.6–19)	0.306	0.2 (−0.1–0.6)	0.228
Skinfold measurements (*n* = 16 boys, *n* = 8 girls)								
Body fat girls (%)	13 (5–16)	14 (6–17)	0.263	11 (6–17)	12 (5–18)	0.715	2 (−0.8–4)	0.200
Body fat boys (%)	9 (8–11)	9 (7–11)	0.496	10 (8–11)	7 (7–10)	0.176	1 (−2–4)	0.439
BOD POD measurements (*n* = 15 boys, *n* = 8 girls)								
Body fat girls (%)	20 (16–24)	27 (21–32)	0.091	23 (17–30)	22 (18–26)	0.893	5 (−2–13)	0.170
Body fat boys (%)	16 (13–22)	16 (13–20)	0.649	13 (9–14)	15 (8–18)	0.208	−3 (−6–1)	0.104
Fat-free mass girls (%)	80 (76–84)	73 (68–79)	0.091	77 (70–83)	78 (76–83)	0.893	−5 (−13–2)	0.170
Fat-free mass boys (%)	84 (78–87)	84 (80–87)	0.649	87 (86–91)	87 (82–92)	0.208	3 (−1–6)	0.104
Consult dietician								
% of predicted REE^a^	112 (103–119)	114 (106–123)	0.158	111 (99–121)	111 (104–123)	0.272	2 (−8–12)	0.689
Protein intake (g)^b^	51 (42–64)	63 (55–69)	**0**.005*	59 (45–76)	50 (43–60)	0.722	15 (0.4–30)	**0**.044*
Absolute difference caloric intake and TEE (calories)^b^	649 (260–992)	364 (47–590)	0.058	432 (141–665)	586 (233–902)	0.248	−388 (−674–−102)	**0**.008*
caloric intake as % of TEE	82% (63–93)	89% (73–101)		84% (69–99)	75% (58–93)			

SDS, standard deviation score.

BOD POD measurements were missing (before or after exercise) in four children due to equipment failure.

Data are presented as median (IQR). Differences over the exercise period and control period were analyzed using the Wilcoxon signed ranks test. A generalized equations approach model was used to compare change over the control to the exercise period (described as the effect size including 95% CI and matching *p*-value). Difference between the baseline MFS outcomes and healthy population was measured using the Wilcoxon one sample test.

Bold and * values mean *p* < 0.05.

^a^ *n* = 24 over the exercise period as in three patients the REE measurements failed.

^b^ *n* = 12 patients properly filled in a food diary before and after the control period, and *n* = 19 patients properly filled in a food diary before and after exercise.

## Discussion

This study is the first to investigate the effects of a lifestyle intervention, in a—for the population of patients with a Fontan circulation—relatively large group of children/adolescents with a Fontan circulation on fatigue, fear of exercise, and nutritional status.

### Fatigue

Although fatigue is widely investigated in children with various chronic diseases, remarkably few studies have assessed fatigue in children with CHD. We could only identify two previous studies, of which one is recently preprinted (version 1, research square): Vos et al. measured fatigue in 259 Dutch children with various CHD's using the same questionnaire as we did (29). In this study, 42% of the included single ventricle patients (*n* = 33) reported to experience fatigue (calculated by a deviation of at least 1 SD from healthy peers) (29). In the study by Bektas et al., 18% of children with a CHD (including seven Fontan patients) reported to have moderate to severe fatigue (30). In our study before training, both self and parent reports demonstrated higher levels of fatigue compared to healthy children on all domains (20). After training, two parent-reported domains improved significantly compared to the control period. The discrepancy between the parent and child reports might in part be explained by a lack of power in the child forms (parent reports *n* = 48, child reports *n* = 25). Interestingly, the preprinted study of de Vos et al. showed that only the number of previous surgeries and Watt/kg and VO2/kg measured during cardiopulmonary exercise testing significantly correlated with the level of fatigue in children with CHD (29). After our intervention, both Watt/kg and VO2/kg improved significantly, possibly contributing to the improved parent-reported fatigue.

### Fears

Fear of exercise on the anxiety thermometer was already low for both children and parents before the intervention and decreased even more in children after the intervention compared to the control period. Children may have been nervous to start the exercise intervention or maybe became more aware of the impact of their disease on sports competencies by participating in the study. The only other study measuring the effects of training on fear regarding exercise in patients with a CHD also found a significant reduction in anxiety regarding exercise among adolescents (14).

### Nutritional status

Recently, evidence regarding unfavorable body composition (including high body fat percentages together with a decreased muscle mass) in both pediatric and adult Fontan patients is rising (31–34). Several studies found associations between unfavorable body composition in Fontan patients and adverse outcomes, including decreased exercise capacity and cardiac failure (35). In our study, children had a normal median body mass index (BMI) and body fat percentage measured by BOD POD indicating a lean to moderately lean posture compared to healthy peers (26, 33). Fat percentage measured by fourfold skinfold indicated a body fat percentage below the 50th percentile (25). After training, body fat percentage (and thus fat-free percentage) remained unchanged, which is in contrast to the large improvements we measured in strength of the children. Possibly assessment techniques for body composition were not sensitive enough to measure subtle changes in body composition (as might be expected during a period of only 12 weeks), and sufficient statistical power to measure these small differences is lacking. Also, other body composition measurement techniques such as dual-energy x-ray absorptiometry might be more sensitive; however, this technique is also more expensive and time consuming (36, 37). In addition, currently future studies could include nutritional biomarkers related to muscle mass and fat mass (such as adipocytokines, myokines) to measure exercise effects on nutritional status. Besides some small studies investigating REE in very young Fontan patients, aged 3.6 ± 2.6, immediately after Fontan surgery, no studies have reported upon REE in older Fontan patients before (38). This is remarkable, since it is well known that malnutrition (undernourishment) is a large problem in this population. Malnutrition may lead to longer hospital stays, higher mortality rates, and even worse neurodevelopmental and growth outcomes in the long term (39–42). A recent study by Sekhon et al. even showed that moderate to severe malnutrition is still present in 6.5%–12.9% of patients 10 years after the Fontan completion (43). Measured REE in children included in our study was increased compared to reference values (median 112% of predicted); simultaneously, children consumed a median of 649 calories (260–992) below their predicted TEE, increasing the caloric deficit further. After the intervention, median REE did not change compared to the control period. However, average caloric intake increased (thus caloric deficit decreased) by more than half compared to the control period and amount of protein (g) increased significantly, indicating that patients followed the dietary advice. We could not identify previous studies prescribing a (personalized) diet advice or even investigating the gap between intake and (recommended) TEE in Fontan children who passed the age of 6 years. Patients should be more closely monitored and more research is needed to determine the added value of proper nutritional management in this group, preventing large consequences of malnutrition on various health outcomes later in life.

### Strengths and limitations

Our study has several strengths and limitations. This study is the first to investigate the effects of a lifestyle intervention in a relatively large group of children with a Fontan circulation. Occasionally, investigated outcomes in Fontans were studied including fear regarding exercise, fatigue, and nutritional status. A weakness of our study is the small control group, of 14 patients. Also, researchers in the study could not be blinded during measurements due to the content of the study. Although we found a significant decrease in caloric deficit and increase in protein intake, indicating that patients followed the dietary advice, we cannot verify this with certainty. In addition, eight patients did not fill in the food diary properly (before or after) the intervention, leading to a smaller sample size. This is the first study performing the innovative and non-invasive whole-body densitometry using the BOD POD in Fontan patients to measure body composition. Although research investigating the BOD POD in adult patients claims high test–retest reliability, we occasionally saw large intra-patient differences in patients with mostly stable weight, BMI, and body fat percentage measured by the skinfold method (44, 45).

### Patients experience and recommendations

Overall the 12-week lifestyle intervention positively impacted participating children and their parents. Most children (and their parents) reported not to feel any hesitation to exercise (and let their child exercise) after the intervention. Parents reported their children to be more energetic, and also the children themselves stated that daily life activities including walking stairs and cycling to school took less effort. Besides positive effects on fear and fatigue, many parents reported the tailored diet advice to be very helpful, as most were unaware of the (unintended) caloric deficits of their children. Although measured body composition did not change significantly, children started to feel more muscular after the resistance training in comparison to before the intervention, which was consistent with our own clinical observations. When considering all results, we think pediatric cardiologists should actively encourage children with a Fontan circulation to participate in resistance training (or other fitting sports), and importantly more attention should be paid to proper nutritional management.

## Conclusion

This study is the first to investigate the effects of a 12-week supervised lifestyle intervention in a relatively large group of children with a Fontan circulation on fatigue, fear, and nutritional status. The intervention improved parent-reported fatigue, decreased child-reported fear, and had positive impacts on caloric and protein intake.

## Data Availability

The raw data supporting the conclusions of this article will be made available by the authors, without undue reservation.
