# Supplementary material for: A 12-week lifestyle intervention: effects on fatigue, fear, and nutritional status in children with a Fontan circulation
Source: Front Pediatr. 2023 Nov 6;11:1154015. doi: 10.3389/fped.2023.1154015 (PMC10657862; doi:10.3389/fped.2023.1154015)
Supplement: Supplementary file 1 [file Image1.pdf]

Supplemental data 1 – Fear thermometer

ANGST VOOR SPORT EN BEWEGING

**De "angst-thermometers" hieronder geven aan hoe bang iemand kan zijn om te sporten of flink te bewegen.
Wil jij aub één cirkeltje zetten om de thermometer die het beste aangeeft hoe *bang* jij je *op dit moment* voelt voor *sport en beweging?***

***Je mag maar bij* 1 thermometer een cirkeltje zetten**

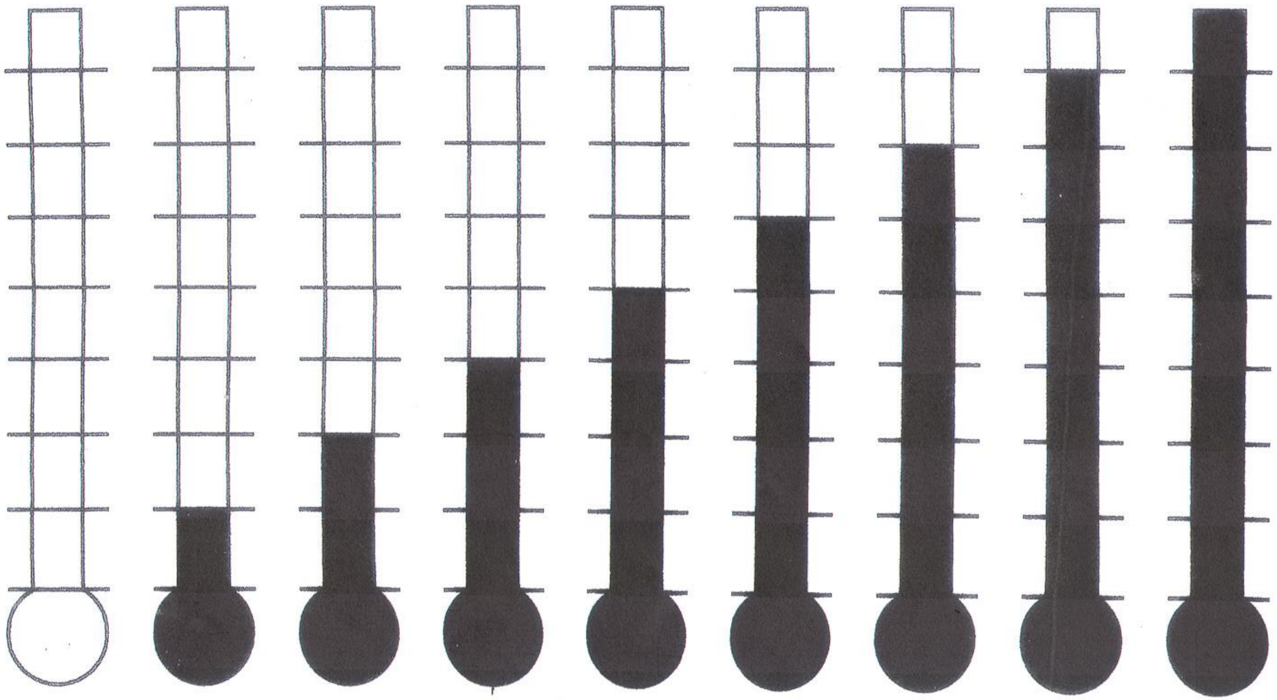

0	2	4	6	8
Helemaal niet	Een klein beetje	Enigszins/Een beetje	Erg	Heel erg
